# Biochemical recurrence after radical prostatectomy according to nadir prostate specific antigen value

**DOI:** 10.1371/journal.pone.0249709

**Published:** 2021-05-03

**Authors:** Jae Hoon Chung, Jae Yong Jeong, Ji Youl Lee, Was Song, Minyong Kang, Hyun Hwan Sung, Hwang Gyun Jeon, Byong Chang Jeong, Seong IL Seo, Hyun Moo Lee, Seong Soo Jeon

**Affiliations:** 1 Department of Urology, Samsung Medical Center, Sungkyunkwan University School of Medicine, Seoul, Republic of Korea; 2 Department of Urology, Kangbuk Samsung Hospital, Sungkyunkwan University School of Medicine, Seoul, Republic of Korea; 3 Department of Urology, College of Medicine, The Catholic University of Korea, Seoul, Republic of Korea; 4 Department of Biomedicine & Health Sciences, The Catholic University of Korea, Seoul, Republic of Korea; University of Sydney, AUSTRALIA

## Abstract

The hypersensitive prostate specific antigen (PSA) test can measure in 0.01 ng/mL units, and its efficacy for screening after radical prostatectomy (RP) has been reported. In this study, we assessed patients who underwent RP to evaluate whether the nadir value affects biochemical recurrence (BCR). From 1995 to 2014, patients classified as N0 who had negative resection margins and a nadir PSA of less than 0.2 ng/mL were evaluated. The characteristics, pathological outcomes, PSA after RP, and BCR were assessed. A total of 1483 patients were enrolled. Among them, 323 (21.78%) patients showed BCR after RP. The mean age of the BCR group was 63.86±7.31 years, and while that of the no-recurrence group was 64.06±6.82 years (*P =* 0.645). The mean preoperative PSA of the BCR group was 9.75±6.92 ng/mL and that of the no-recurrence group was 6.71±5.19 ng/mL (*P* < 0.001). The mean time to nadir (TTN) in the BCR group was 4.64±7.65 months, while that in the no-recurrence group was 7.43±12.46 months (*P* < 0.001). The mean PSA nadir value was 0.035±0.034 ng/mL in the BCR group and 0.014±0.009 ng/mL in the no-recurrence group (*P* < 0.001). In multivariable Cox regression analyses, Gleason score, positive biopsy core percentages, minimal invasive surgery, nadir PSA value, and TTN were independently associated with BCR. The mean BCR occurred at 48.23±2.01 months after RP, and there was a significant difference in BCR occurrence according to the nadir PSA value (*P* < 0.001). A high PSA nadir value and short TTN may predict the risk of BCR after successful RP, aiding the identification of candidates for adjuvant or salvage therapies after RP.

## Introduction

Recent advances in urology have led to better surgical candidate selection and advances in surgical technology, but biochemical recurrence (BCR) still occurs in 15–40% of patients after radical prostatectomy (RP) for the treatment of prostate cancer (PCa) [1]. This BCR is associated with disease progression and cancer-specific mortality in PCa patients [2]. There are several reports of pre- and peri-operative risk factors that can predict post-RP BCR, such as pre-operative prostate specific antigen (PSA) value, Gleason score, and cancer stage [3]. Predicting BCR after RP will facilitate the selection of high-risk patients, so that the timing of adjuvant/salvage treatment is not missed and does not affect cancer-specific survival.

PSA was first introduced by Wang et al. in 1979 [4] and has been used to screen for PCa since late 1980 [5]. PSA can not only screen for PCa, it can monitor disease progression in untreated patients and evaluate the response of PCa to treatment. Moreover, PSA is useful for detecting residual and recurrent tumors after definite treatment for PCa such as RP [5–9]. Many assays that measure PSA levels have been developed to date, and ultrasensitive PSA tests that can measure PSA levels below 0.1 ng/mL are widely used [10]. However, debate persists about the clinical utility of ultrasensitive PSA. Ultrasensitive PSA tests have been reported to predict the biochemical recurrence after RP [11–13]. Other studies have reported that ultrasensitive PSA tests onl provide cause PCa patients anxiety without clinical significance [14].

The purpose of this study was to evaluate the association between the nadir value and the occurrence of BCR in RP patients with PCa who had a negative surgical margin and whose PSA value fell under 0.2 ng/mL.

## Materials and methods

### Patients

From January 1995 to December 2014, we retrospectively reviewed patients who underwent RP for PCa. Patients whose nadir value was not less than 0.2 ng/mL, for whom surgical margin involvement was excluded, or without BCR who had a follow-up period less than 36 months were excluded from this study.

### Clinicopathological parameters

To evaluate the patients’ baseline characteristics, age, body mass index, hypertension (HTN), diabetes mellitus (DM), serum PSA, prostate volume (measured by transrectal ultrasonography or magnetic resonance imaging), PSA density (PSAD), results of pre-operative biopsy (including Gleason score, positive core percentage, tumor volume percentage), and clinical stage were evaluated. Peri- and post-operative outcomes, including operative times, estimated blood loss, operation type, pathological outcomes, pathologic stage, nadir PSA value, time to nadir (TTN), and follow-up periods, were also assessed.

### PSA follow-up

In this study, the PSA test was performed using ADVIA Centaur^®^ PSA assay(Siemens, Muenchen, Germany), which measures total PSA concentrations up to 100 ng/mL with a minimum detectable concentration of 0.01 ng/mL. Analytical sensitivity is defined as the concentration of total PSA that corresponds to the relative light units that are two standard deviations greater than the mean relative light units of 20 replicate determinations of the PSA zero standard.

### Statistical analysis

The groups were compared using the chi-square test for categorical variables and the Student’s *t*-test for continuous variables. To assess the hazard ratio of risk factors for BCR, univariable and multivariable Cox regression analyses were performed [15]. The occurrence of BCR by nadir value was evaluated using a Kaplan-Meier curve. For the statistical hypothesis tests a significance level of alpha = 0.05 was used for all test [16].

### Ethics statement

This study was performed in agreement with applicable laws and regulations, good clinical practices, and ethical principles as described in the Declaration of Helsinki. The Institutional Review Board of Samsung Medical Center approved the present study (approval no. 2020-02-038-001). Informed consent was waived by the Board. Registered patient information was extracted only from the Samsung Medical Center, Seoul, Korea. All data were analyzed after anonymization and data were collected on a monthly basis.

## Results

In this study, 1483 patients were analyzed. Among them, 323 were identified as having BCR. The patients had an average follow-up period of 91.60 ± 38.24 months postoperatively. All patients had a negative surgical margin and less than 0.2 ng/mL of PSA after RP, and were evaluated for proper resection. None of the enrolled patients received adjuvant treatment before BCR diagnosis.

In terms of baseline characteristics, there was no significant difference in age, BMI, or DM between the BCR and non-BCR groups, but HTN was 27.55% in the BCR group and 32.41% in the non-BCR group (*P =* 0.019). The BCR group showed significant differences in pre-operative parameters including pre-operative PSA, PSAD, biopsy Gleason scores, positive core percentages, tumor volume percentages, and clinical stage compared to the non-BCR group (Table 1).

**Table 1:** Baseline characteristics of patients in the biochemical recurrence versus no-recurrence groups.

	Biochemical recurrence (n *=* 323)	No recurrence (n *=* 1160)	*P* value
Age, years	63.86±7.31	64.06±6.82	0.645
BMI, kg/m^2^	24.63±2.64	24.64±4.80	0.941
HTN, *n* (%)	89 (27.55)	376 (32.41)	0.019
DM, *n* (%)	29 (8.98)	105 (9.05)	0.751
PSA, ng/mL	9.75±6.92	6.71±5.19	<0.001
Prostate volume, mL	31.17±14.31	34.14±15.51	0.002
PSA density, ng/mL^2^	0.35±0.26	0.23±0.21	<0.001
Gleason score, primary	3.52±0.57	3.16±0.43	<0.001
Gleason score, secondary	3.58±0.60	3.32±0.54	<0.001
Gleason score	7.07±0.95	6.51±0.84	<0.001
Positive core, %	40.45±22.35	26.47±20.15	<0.001
Tumor volume, %	48.73±28.73	32.86±26.25	<0.001
Clinical T stage, *n* (%)			0.014
T1	28 (8.67)	126 (10.86)	
T2	191 (59.13)	762 (65.69)	
T3a	82 (25.39)	219 (18.88)	
T3b	22 (6.81)	53 (4.57)	
Clinical N stage, *n* (%)			0.025
N0	303 (93.81)	1118 (96.38)	
N1	4 (1.24)	16 (1.38)	
Nx	16 (4.95)	25 (2.16)	

Abbreviations: BMI, body mass index; HTN, hypertension; DM, diabetes mellitus; PSA, prostate specific antigen; ASA, American Society of Anesthesiologists.

The mean operation time was 247.03±95.78 minutes for the BCR group and 252.06±101.35 minutes for the non-BCR group (*P =* 0.418). Open surgery was performed more frequently in the BCR group (*P* < 0.001), and its nerve-sparing rate was lower than that of the non-BCR group (*P* < 0.001). Estimated blood loss was 534.86±556.82 mL in the BCR group and 427.42±538.39 mL in the non-BCR group (*P =* 0.002). Pathologic findings and clinical stages in the BCR group were significantly inferior to those in the BCR group. Mean post-operative nadir PSA values were 0.035±0.034 ng/mL in the BCR group and 0.014±0.009 ng/mL in the non-BCR group (*P* < 0.001). The mean follow-up period was 109.10±47.21 months in the BCR group and 86.73±33.78 months in the non-BCR group (*P* < 0.001) (Table 2).

**Table 2:** Operative and post-operative outcomes of the biochemical recurrence versus no recurrence groups.

	Biochemical recurrence (n *=* 323)	No recurrence (n *=* 1160)	*P* value
Operation time, mins	247.03±95.78	252.06±101.35	0.418
Estimated blood loss, mL	534.86±556.82	427.42±538.39	0.002
Operation type, n (%)			<0.001
RPP	113 (34.98)	298 (25.69)	
RRP	66 (20.43)	159 (13.71)	
LRP	16 (4.95)	106 (9.14)	
RALP	128 (39.63)	597 (51.47)	
Nerve-sparing, *n* (%)			<0.001
None	162 (50.15)	383 (33.02)	
Unilateral	79 (24.46)	286 (24.66)	
Bilateral	74 (22.91)	485 (41.81)	
Gleason score, primary	3.50±0.54	3.17±0.42	<0.001
Gleason score, secondary	3.76±0.69	3.63±0.60	0.002
Gleason score	7.26±0.87	6.81±0.77	<0.001
T stage, *n* (%)			<0.001
T0	1 (0.31)	8 (0.69)	
T2a	34 (10.53)	258 (22.24)	
T2b	7 (2.17)	12 (1.03)	
T2c	142 (43.96)	676 (58.28)	
T3a	108 (33.44)	184 (15.86)	
T3b	31 (9.60)	22 (1.90)	
N stage, *n* (%)			<0.001
Nx	259	1056	
N0	64	104	
Nadir PSA, ng/mL	0.035±0.034	0.014±0.009	<0.001
Time to nadir, months	4.64±7.65	7.43±12.46	<0.001
Follow up, months	109.10±47.21	86.73±33.78	<0.001

Abbreviations: BMI, body mass index; HTN, hypertension; DM, diabetes mellitus; PSA, prostate specific antigen; ASA, American Society of Anesthesiologists.

Factors that independently influenced BCR from the multivariable Cox regression analysis were biopsy Gleason score (hazard ratio [HR], 1.414; *P =* 0.012), positive core percentages (HR, 1.017; *P =* 0.002), minimally invasive surgery (HR, 0.491; *P =* 0.002), final Gleason score (HR, 1.391; *P =* 0.037), pathologic stage T3b (HR, 1.283; *P =* 0.014), nadir PSA (HR, 1.254; *P* < 0.001), and TTN (HR, 0.917; *P* < 0.001) (Table 3).

**Table 3:** Univariable and multivariable Cox regression analyses of biochemical recurrence.

	Univariable			Multivariable		
	HR	95% CI	*P* value	HR	95% CI	*P* value
Age	0.996	0.978–1.014	0.645			
BMI	0.999	0.971–1.028	0.957			
HTN	0.713	0.536–0.947	0.019	0.887	0.551–1.428	0.622
DM	0.932	0.603–1.440	0.751			
PSA	1.085	1.062–1.109	<0.001	1.058	0.979–1.145	0.156
Prostate volume	0.986	0.976–0.995	0.003	0.991	0.971–1.011	0.375
PSA density	8.56	4.831–15.168	<0.001	0.524	0.060–4.593	0.559
Biopsy Gleason score	2.016	1.749–2.325	<0.001	1.414	1.078–1.856	0.012
Positive core, %	1.028	1.022–1.034	<0.001	1.017	1.006–1.028	0.002
Tumor volume, %	1.02	1.016–1.025	<0.001	1.002	0.994–1.011	0.592
Clinical stage						
T1	1	reference	-			
T2	1.128	0.727–1.750	0.591			
T3a	1.685	1.041–2.728	0.034	1.131	0.500–2.556	0.768
T3b	1.868	0.981–3.557	0.057	1.26	0.416–3.811	0.683
Operation type						
Open	1	reference	-			
MIS	0.523	0.408–0.671	<0.001	0.491	0.314–0.767	0.002
Nerve-sparing						
None	1	reference	-			
Unilateral	0.653	0.479–0.890	0.007	0.859	0.519–1.422	0.555
Bilateral	0.361	0.266–0.490	<0.001	0.667	0.406–1.094	0.667
Pathology Gleason score	1.755	1.530–2.014	<0.001	1.391	1.020–1.897	0.037
Pathologic stage						
T0	1	reference	-			
T2a	1.054	0.128–8.690	0.961			
T2b	4.667	0.478–45.546	0.185			
T2c	1.68	0.209–13.542	0.626			
T3a	4.696	0.579–38.055	0.147			
T3b	11.273	1.314–96.722	0.027	1.283	1.052–1.566	0.014
Nadir PSA, ng/mL	3.877	3.446–4.363	<0.001	1.254	1.199–1.311	<0.001
Time to nadir, months	0.955	0.934–0.976	<0.001	0.917	0.882–0.953	<0.001

Abbreviations: BMI, body mass index; HTN, hypertension; DM, diabetes mellitus; PSA, prostate specific antigen; ASA, American Society of Anesthesiologists, MIS minimal invasive surgery.

**Fig 1:**
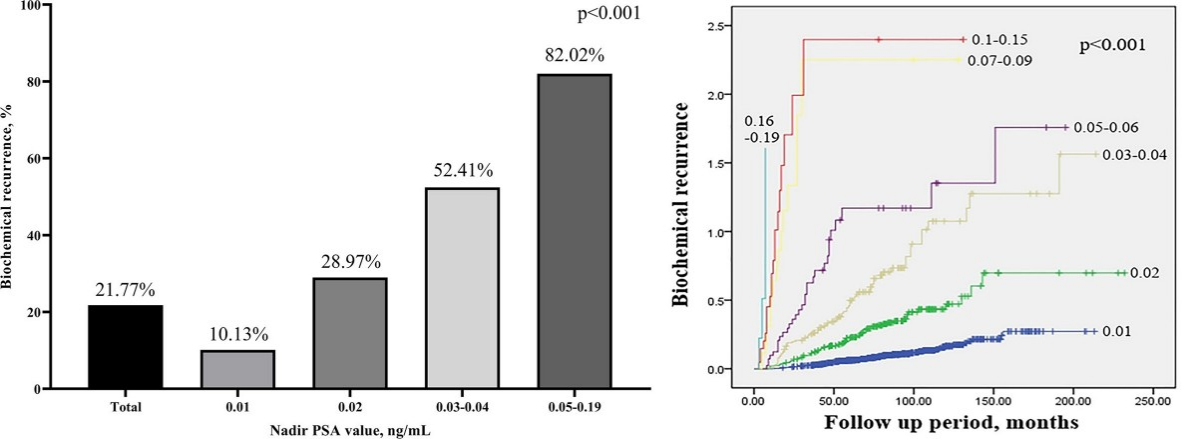
Biochemical recurrence after radical prostatectomy by nadir prostate specific antigen level. Chi-square test, Kaplan-Meier curve of biochemical recurrence by nadir prostate specific antigen value.

**Fig 2:**
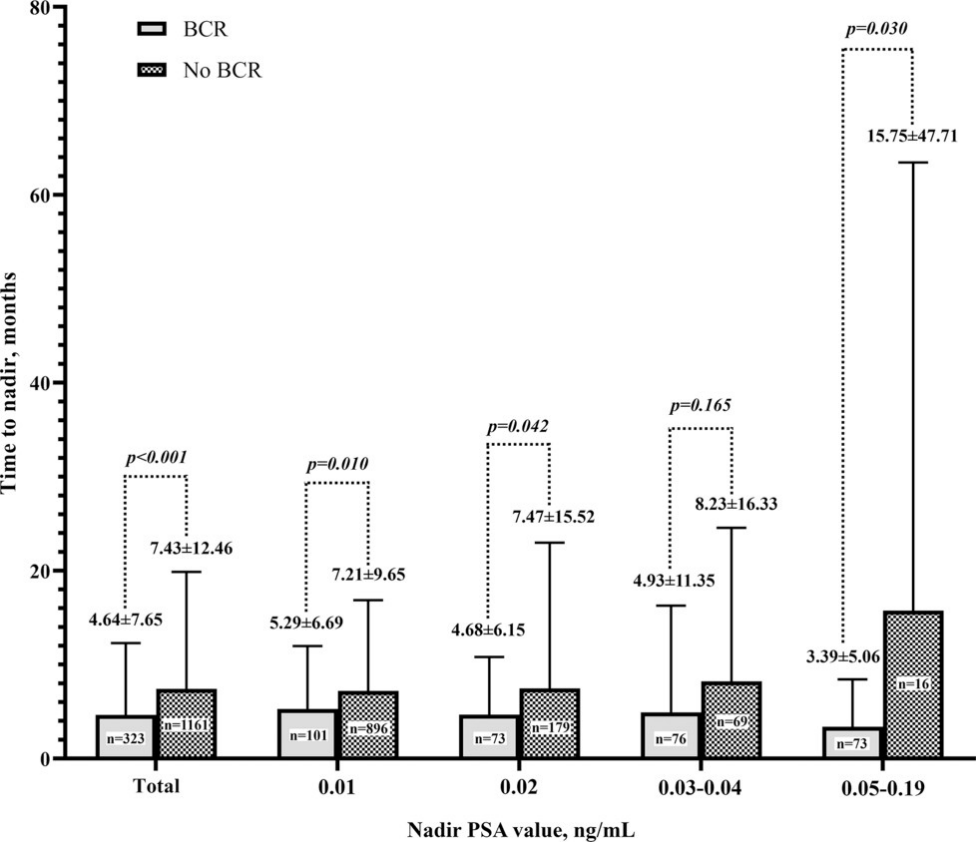
Comparison of time to nadir of biochemical recurrence (BCR) group versus no-recurrence groups using Student’s t-test.

BCR occurred in 21.77% of all RP patients, 10.13% in patients with a nadir PSA value of 0.01 ng/mL, and 28.97% in patients with 0.02 ng/mL. BCR also occurred in 52.41% of patients with a nadir PSA value of 0.03–0.04 ng/mL and 82.02% of patients with a nadir PSA value of 0.05–0.19 ng/mL (Fig 1). As time passed, the nadir PSA value increased and the occurrence of BCR was significantly higher (*P* < 0.001) (Fig 1). Mean TTN was 4.64±7.65 months in the BCR group and 7.43±12.46 months in the non-BCR group (*P* < 0.001). Considering a nadir PSA value of 0.01 ng/mL, the mean TTN of BCR patients was 5.29±6.69 months, while that of non-BCR patients was 7.21 ± 9.65 months (*P =* 0.010). Among patients with a nadir PSA value of 0.02 ng/mL, the mean TTN was 4.68±6.15 months in the BCR patients and 7.47±15.52 months in those with non-BCR (*P =* 0.042). Among patients with a nadir PSA value of 0.03–0.04, the mean TTN was 4.93±11.35 months in the BCR group and 8.23±16.33 months in the non-BCR group (*P =* 0.165). Among patients with a nadir PSA value of 0.05–0.19, the mean TTN was 3.39±5.06 in the BCR group and 15.75±47.71 months in the non-BCR group (*P =* 0.030) (Fig 2).

## Discussion

The results showed that a higher nadir PSA value could reflect increasing BCR risk after RP. Moreover, a longer TTN tended to reduce the risk of BCR after successful RP, and a PSA elevation is the first sign of recurrent PCa after RP. Moreover, PSA failure is inevitable with prostate cancer-specific mortality [17]. Since the first-generation PSA assay was measured at 0.3–0.6 ng/mL, it was not suitable for clinical use [9,18]. However, due to advances in technology, PSA measurements have been made below 0.2 ng/mL, and the American Urological Association and the European Association of Urology panel have defined BCR as a value above 0.2 ng/mL [19]. Ultrasensitive PSA assays have been developed that measure values below 0.1 ng/mL. The 0.001 ng/mL value has also been measured recently, but the definition of BCR is still used [20].

There are reports that the PSA value measured at a certain time after RP is associated with the risk of developing BCR [21–23]. Kang et al. reported that a PSA value greater than 0.03 ng/mL at 3 months after RP increases the risk of BCR [23]. The results of present study also showed a rapid increase in BCR when the nadir PSA value was over 0.03. Some reports suggest that the re-establishment of BCR criteria is required using the recently used ultrasensitive PSA assay [24]. If these contents are established through large scale, prospective, and long-term studies, it may be possible to reduce the cancer-specific mortality rate by enabling appropriate adjuvant treatment.

The present study did not confirm the nadir PSA value at a specific time point after RP; rather, it evaluated the lowest value during the follow-up period as the nadir value. These results suggest that a longer TTN tends to reduce the incidence of BCR. This is in stark contrast to reports that higher PSA levels at 1 or 3 months after RP increase the risk of BCR. This confirms, the importance of the final nadir PSA value. Moreover, additional factors that can predict BCR after RP, such as TTN and final nadir PSA value may be helpful for patient consultancy after RP. In contrast to the present study, Skove et al reported that a TTN of less than 3 months after RP lowers the risk of BCR compared to a TTN of 3–6 months after RP [21]. Moreover, Chung et al. reported that, among patients with a nadir PSA exceeding 0.9 ng/mL, a prolonged TTN increases PCa-specific mortality [25]. Unlike in previous studies, in the present study, the nadir PSA value was not determined at a specific time after RP and the lowest value was assessed during the follow-up period. In addition, we analyzed the patients whose PSA level was less than 0.2 ng/mL after RP. The mean follow-up period of 92 months is sufficient to assess the association between nadir PSA value, TTN, and BCR. Moreover, we evaluated TTN according to the presence or absence of BCR for each nadir PSA value, assuming that the TTN could be shortened if the nadir value was relatively high.

This result is expected to provide more useful data for clinical practice. Previous studies suggested that close observation of high-risk patients will be possible if the PSA confirmed at a certain time after surgery can predict the occurrence of BCR. However, oncologists require close observation of every patient during cancer management. Even if successful RP has been performed for the treatment of PCa, the risk of BCR must be considered and periodic PSA tests and radiological examinations are required [26]. After the complete resection of prostate cancer, low nadir PSA value and a longer TTN suggest a better prognosis.

We confirmed that the risk of BCR was higher in patients with a high tumor burden and high cancer stage as reported long-term follow-up study [27]. The present study showed that other risk factors for BCR were Gleason score, positive core percentage, and pathologic stage. This study also confirmed that high-risk prostate cancer increased the risk of BCR.

In this study, minimally invasive surgery (MIS) reduced the risk of BCR. However, there are many reports suggest that MIS dose not have no different oncological outcomes, such as BCR, compared with open surgery [28,29]. However, in most previous studies, the definition of BCR is not clear, and there is a paucity of quality trials. According to Coughlin et al., MIS showed lower BCR than open RP in a randomized controlled trial [30]. These results were conservatively interpreted, but the possibility that MIS could reduce the risk of BCR was suggested. Although it was not possible to report the mechanism by which MIS reduces the risk of BCR, our results can be considered valid because the present study was relatively large and examined long-term follow-up data using a strict BCR definition (PSA of 0.2 ng/mL).

The main limitation of this study is that it did not evaluate salvage treatment after BCR or cancer-specific mortality. In addition, the absence of an evaluation of neoadjuvant androgen deprivation therapy (ADT) may have acted as a bias. However, none of the patients underwent RP after neoadjuvant ADT in advanced PCa and none maintained post-operative ADT. Moreover, because this was retrospective study, the follow-up schedule after RP was not uniform. However, this study’s findings may be of interest since it analyzed risk factors of BCR only in patients whose PSA was determined to be a successful resection at less than 0.2 ng/mL after RP.

## Conclusions

Advanced cancer stage, high tumor volume, and open surgery are evaluated as risk factors for BCR. Moreover, a higher nadir PSA value and shorter TTN increases the risk of BCR after successful RP.

## Supporting information

S1 File (XLS)
