# Correction: Biochemical recurrence after radical prostatectomy according to nadir prostate specific antigen value

**DOI:** 10.1371/journal.pone.0269908

**Published:** 2022-06-08

**Authors:** Jae Hoon Chung, Jae Yong Jeong, Ji Youl Lee, Wan Song, Minyong Kang, Hyun Hwan Sung, Hwang Gyun Jeon, Byong Chang Jeong, Seong IL Seo, Hyun Moo Lee, Seong Soo Jeon

The fourth author’s name is spelled incorrectly. The correct name is: Wan Song. The correct citation is: Chung JH, Jeong JY, Lee JY, Song W, Kang M, Sung HH, et al. (2021) Biochemical recurrence after radical prostatectomy according to nadir prostate specific antigen value. PLOS ONE 16(5): e0249709. https://doi.org/10.1371/journal.pone.0249709
